# Editorial: Advanced nanomaterials for bio-derived diagnostics and therapy

**DOI:** 10.3389/fbioe.2023.1277107

**Published:** 2023-08-23

**Authors:** Hongcheng Sun, Syed Mazhar Shah, Yuan Gao

**Affiliations:** ^1^ College of Material Chemistry and Chemical Engineering, Key Laboratory of Organosilicon Chemistry and Material Technology, Ministry of Education, Hangzhou Normal University, Hangzhou, Zhejiang, China; ^2^ Department of Natural Sciences and Humanities, (RCET), University of Engineering and Technology, Lahore, Lahore, Pakistan; ^3^ State Key Laboratory on Integrated Optoelectronics, Key Laboratory of Gas Sensors, College of Electronic Science and Engineering, Jilin University, Changchun, Jilin, China

**Keywords:** biosensors, therapy, phototherapy, bioimaging, drug delivery, anti-bacteria, antitumor

Nowadays, with the growing attention on personal healthcare, much more emphasis is placed on the early diagnosis and treatment of disease. Advanced nanomaterials and nanotechnologies have been the key driving force in modern medical diagnosis and treatment. The growing demand of advanced nanomaterials and biotechnology presents unprecedented opportunities and challenges because of their unique physical/chemical properties. Some interesting nanomaterials or nanostructures are helpful for the revolutionizing of the current methods and levels of detection, imaging, diagnosis, and treatment of diseases. Therefore, the development of new materials for the early diagnosis of diseases or precision therapy is urgently needed. In this Research Topic, the published works are mainly focused on advanced nanomaterials for cancer therapy. The multidisciplinary attempts have led to the development of several exciting nanotherapeutics, which might provide a technical support for the advancement of sophisticated nanomaterials for the bio-derived diagnostics and therapy of disease.

Rational design for advanced nanostructures or nanomaterials is a promising solution for improving the therapeutic efficacy. Nanostructures offer significant advantages as they may overcome long-term Research Topic such as poor drug solubility, drug metabolism, systemic half-life, and undesirable side effects (Yu et al., 2023). However, the challenge still remains regarding how to enhance permeability and retention in the tumor stroma. During the recent decades, researchers have developed numerous kinds of nanostructures with different size, shapes, or even charge distributions to enhance the accumulation of therapeutic agents in tumor sites (Sun et al., 2017). Not only that, strategies like surface modification with specific ligands, camouflage with cell membranes, and charge or size reversal triggered by the tumor-microenvironment (TME) have also been employed to improve their clinical potential.

However, there is a significant need for the additional validation of the biological composition and activity of these biomimetic nanostructures to ensure their real properties *in vivo*. Ferreira et al. developed a design of experiments (DoE) strategy for the first time to optimize the development of cell membrane-coated nanostructures for cancer therapy (Ferreira et al.). A fractional two-level three-factor design of experiments, including the ratio, method of coating procedure, and time/cycles applied on the responses of size, zeta potential, and polydispersity index, was then applied to optimize the coating of nanosystems, which was referred to DoE optimization. The results revealed the associated techniques could provide the ideal structure for obtaining the desired properties with improved specific targeting to homotypic tumor cells. This work will provide deeper insight and comprehensive understanding regarding the structure-to-property correlation and the development of optimized biomimetic nanostructures for advanced bio-derived diagnostics and therapy.

Moreover, phototherapy including photodynamic therapy (PDT), photothermal therapy (PTT), and photoimmunotherapy (PIT) is a newly developed modality for cancer therapy because of their precise controllability, high selectivity, non-invasiveness, and negligible drug resistance. The integration of photoinduced diagnostic imaging and concurrent *in situ* therapy into a single formulation with precise spatial co-localization toward tumor treatment has been exploited and proved to be a preeminent protocol of cancer theranostics, which is referred to as phototheranostics. Recently, inorganic nanoparticles or molecular fluorophores with precise molecular engineering were used as phototheranostic agents toward multimodal imaging-guided synergistic therapy (Yang et al., 2021). It was revealed the multimodal treatment in combination with precise diagnostics can produce a remarkable superadditive (1 + 1 > 2) effect.

Inspired by this, Peng et al. reported a new research work to construct a novel hollowed mesoporous selenium oxide (hmSeO_2_) platform for simultaneous near infrared II (NIR II) fluorescence imaging and synergistic breast carcinoma therapy (Peng et al.). After encapsulation with a photosensitizer (ICG) and surface modification with tumor targeting peptides, the constructed nanoplatform (hmSeO_2_@ICG-RGD) facilitated targeted delivery to tumor tissues via systemic administration. Meanwhile, after being degraded in the acidic tumor microenvironment (pH 6.5), ICG could not only act as an NIR II contrast agent which discriminates tumor outlines, but also laser-triggered photothermal effects could facilitate the generation of ROS “storms” via SeO_2_ nanogranules for significant tumor cell killing, which represent a potential diagnostic and therapeutic nanoplatform for clinical application.

Bacterial therapy has received widespread attention in recent years, which originated from the discovery two hundred years ago that bacterial infections can cure malignant tumors to some extent. Because of the hypoxic surroundings of solid tumor tissue, obligate anaerobe or facultative anaerobic organisms (such as *Escherichia coli* and *Salmonella typhimurium*) can selectively enrich in the tumor area for self-proliferation. Researchers have developed various drug delivery systems using engineered bacteria to overcome the targeted deficiency of traditional tumor therapeutic drugs, pharmacokinetic defects, and drug resistance caused by hypoxic microenvironment. Researchers have constructed “trojan nanobacteria” through the combination of bacteria with anti-cancer drugs or nanomaterials to jointly improve the anti-tumor effect with the tumor targeting of bacteria and the efficacy of drugs (Sun et al., 2022). On the other hand, with the self-reproduction and biological expression characteristics of living bacteria, *de novo* bacterial therapy strategies were employed through expressed toxic proteins or activated tumor immunotherapy. Therefore, it can be seen that engineered bacteria are an ideal platform for anti-tumor therapy (Chen et al., 2022).

Building upon this foundation, combined with the advantages of bacteria therapy and micromotors, Du et al. proposed the ultrasound-responsive bacteria-driven biohybrid microbot for targeted drug delivery and controlled release. Doxorubicin and perfluoro-n-pentane were encapsulated in polylactic acid-glycolic acid, which was further bonded to the surface of *E. coli* MG1655 through amide reaction to create the ultrasound-responsive SonoBacteriaBot (Du et al.). Interestingly, the SonoBacteriaBot can be accumulated in tumor regions via bacterial self-propulsion without causing harm to critical organs. Meanwhile, it can enhance the signal of ultrasound imaging after ultrasound irradiation and release, which has significant potential applications for therapeutic drug delivery in clinical settings.

In summary, the series of articles published in this Research Topic introduces the latest strategies to develop advanced nanomaterials for the bio-derived therapy of tumors. The research papers in this Research Topic involve the optimization of traditional nanomedicines using the DoE strategy, the development of all-in-one nanomedicine with integrated diagnosis and treatment, and the establishment of self-propelled bacterial micromotors for precise cancer therapy. *In vitro* and *in vivo* research results and analysis will provide valuable templates for endowing the development and application of advanced nanomaterials for clinical diagnostics and therapy.
